# Varicella Zoster Virus infects mucosal associated Invariant T cells

**DOI:** 10.3389/fimmu.2023.1121714

**Published:** 2023-03-17

**Authors:** Shivam K. Purohit, Alexandra J. Corbett, Barry Slobedman, Allison Abendroth

**Affiliations:** ^1^ Infection, Immunity and Inflammation, School of Medical Sciences, Faculty of Medicine and Health, Charles Perkins Centre, University of Sydney, Sydney, NSW, Australia; ^2^ Department of Microbiology and Immunology, The University of Melbourne, at The Peter Doherty Institute for Infection and Immunity, Melbourne, VIC, Australia

**Keywords:** Varicella Zoster Virus, productive infection, MAIT cells, herpesvirus, innate-like T cells

## Abstract

**Introduction:**

Mucosal Associated Invariant T (MAIT) cells are innate-like T cells that respond to conserved pathogen-derived vitamin B metabolites presented by the MHC class I related-1 molecule (MR1) antigen presentation pathway. Whilst viruses do not synthesize these metabolites, we have reported that varicella zoster virus (VZV) profoundly suppresses MR1 expression, implicating this virus in manipulation of the MR1:MAIT cell axis. During primary infection, the lymphotropism of VZV is likely to be instrumental in hematogenous dissemination of virus to gain access to cutaneous sites where it clinically manifests as varicella (chickenpox). However, MAIT cells, which are found in the blood and at mucosal and other organ sites, have yet to be examined in the context of VZV infection. The goal of this study was to examine any direct impact of VZV on MAIT cells.

**Methods:**

Using flow cytometry, we interrogated whether primary blood derived MAIT cells are permissive to infection by VZV whilst further analysing differential levels of infection between various MAIT cell subpopulations. Changes in cell surface extravasation, skin homing, activation and proliferation markers after VZV infection of MAIT cells was also assessed via flow cytometry. Finally the capacity of MAIT cells to transfer infectious virus was tested through an infectious center assay and imaged via fluorescence microscopy.

**Results:**

We identify primary blood-derived MAIT cells as being permissive to VZV infection. A consequence of VZV infection of MAIT cells was their capacity to transfer infectious virus to other permissive cells, consistent with MAIT cells supporting productive infection. When subgrouping MAIT cells by their co- expression of a variety cell surface markers, there was a higher proportion of VZV infected MAIT cells co-expressing CD4+ and CD4+/CD8+ MAIT cells compared to the more phenotypically dominant CD8+ MAIT cells, whereas infection was not associated with differences in co-expression of CD56 (MAIT cell subset with enhanced responsiveness to innate cytokine stimulation), CD27 (co-stimulatory) or PD-1 (immune checkpoint). Infected MAIT cells retained high expression of CCR2, CCR5, CCR6, CLA and CCR4, indicating a potentially intact capacity for transendothelial migration, extravasation and trafficking to skin sites. Infected MAIT cells also displayed increased expression of CD69 (early activation) and CD71 (proliferation) markers.

**Discussion:**

These data identify MAIT cells as being permissive to VZV infection and identify impacts of such infection on co- expressed functional markers.

## Introduction

Compared to conventional T lymphocytes that can respond to a very wide array of peptide antigens, innate T cells react to conserved antigenic patterns either pathogenically or host derived. Mucosal Associated Invariant T (MAIT) cells are one of the largest human innate T cell subpopulations, representing approximately 3% of all circulating T cells and up to 45% of liver T cells in healthy donors (1, 2). MAIT cells express a limited semi-invariant T cell receptor (TCR) repertoire allowing for their recognition of microbial metabolite neo-antigens presented by the monomorphic MHC- related 1 (MR1) molecule (3–6). The most well characterized MAIT cell agonist ligand, 5-(2-oxopropylideneamino)-6-_D_-ribitylaminouracil (5-OP-RU), is a pyrimidine derivate from the vitamin B2 (riboflavin) biosynthesis pathway presented by MR1 (5, 7). The conservation of this biosynthetic pathway across a diverse range of bacterial and fungal species drives evolutionary conservation of the MR1-MAIT cell axis within mammals (8).

Given the inherent barrier surveillance functionality of MAIT cells, they are tailored to have a highly expressed extravasation program mediated by C-C Chemokine receptor (CCR)2, CCR5 and CCR6 expression (9), complimented by their migrational predilection to anatomical sites such as liver, respiratory mucosa and skin mediated by expression of CCR6, CCR5, CCR9, C-X-C Chemokine receptor (CXCR)6 and cutaneous lymphocyte antigen (CLA) (1, 2, 10, 11). Whilst MAIT cells can rapidly react to their cognate ligands, the concomitant delivery of accessory signals such as Toll-Like receptors (TLRs) and cytokines such IL-12 and IL-18 elicit a broader and more sustained effector response (12–16). Indeed, MAIT cells can be solely activated by cytokines in the absence of TCR engagement driving a distinct effector response (17). Co-incubation of MAIT cells with intact riboflavin synthesizing bacteria rapidly stimulates a Tc17-like RORγt driven response characterized by granzyme B mediated cytolytic activity, proinflammatory cytokine expression of IFN-γ, TNF-α, IL-17α, as well as tissue repair signals such as TGF-β and furin (16, 18–20). Comparatively, IL-12 and IL-18 driven activation of MAIT cells is delayed and Tc1-like as characterized by increased T-bet, IFN-γ and granzyme B expression (20). Combined, both modes of MAIT cells activation support a polyfunctional T cell population that is able to enact diverse and distinct effector responses dependent on the micro-environmental cues.

Cytokine driven activation of MAIT cells has stimulated a burgeoning interest in the importance of MAIT cells in controlling viral infections. Whilst a growing body of literature has reported a protective role of MAIT cells in several viral infections both *in vitro* and *in vivo* (21–25), there is a dearth of studies that examines virus infection of MAIT cells. To date, there is only a single study reporting virus infection of MAIT cells; infection and apoptosis of MAIT cells *in vitro* by measles virus (MV) (26).

Varicella Zoster Virus (VZV) is a lymphotropic, highly seroprevalent alpha herpesvirus that causes varicella during primary infection and herpes zoster following reactivation from latency (27). Following exposure to infected respiratory droplets, VZV initially infects the epithelial cells and resident dendritic cells (DCs) lining the upper respiratory tract before gaining access to local lymphoid structures such as the tonsils (28). Here, the transfer of virus is believed to occur from DCs to mature T lymphocytes (28, 29) that express skin homing markers, with several reports characterizing how this enables VZV to reach the host’s cutaneous sites (30–34). We reported that VZV productively infects human natural killer (NK) cells *in vitro*, resulting in their upregulation of skin homing capacity, yet overall functional paralysis (35, 36). These findings suggest that VZV has evolved the capacity to infect a broad range of immune cell types to enhance virus dissemination.

We have previously demonstrated a profound disruption of the MR1 antigen presentation pathway mediated by VZV, thereby suggesting a virally pathogenic importance in abrogating the TCR dependent activation and effector response of MAIT cells (37). However, the direct infection or interaction of VZV with MAIT cells themselves had yet to be investigated, despite the substantial enrichment of MAIT cells in blood as well as migrational proclivity to tissues central to VZV pathogenesis such as airway epithelia and skin. In the current study, we examined the ability of VZV to infect human blood-derived MAIT cells *in vitro.* We demonstrate that VZV productively infects MAIT cells, resulting in a capacity to transmit virus to other cells. VZV infection of MAIT cells was associated with retention or upregulation of markers of extravasation, skin homing potential and/or activation and proliferation. Overall, this study illuminates an innate-like T cell population that can be directly targeted by VZV.

## Materials and methods

### Blood samples and MAIT cell isolation

Healthy adult human donor buffy coats were obtained from Australian Red Cross Lifeblood service from which peripheral blood mononuclear cells (PBMCs) were isolated through density gradient centrifugation using Ficoll-Paque PLUS (GE Healthcare). There was no selection bias based on gender. Isolated PBMCs were cultured in complete RPMI medium (RPMI 1640 with L-Glutamine (Lonza) supplemented with 10% human serum (Sigma-Aldrich). In experiments which utilized purified MAIT cells, MAIT cells were FACS isolated from PBMCs after positive co-staining in FACS buffer (PBS supplemented with 1% FCS and 10 mM EDTA) with fluorochrome conjugated antibodies: anti-CD3, anti-TCR Vα7.2 and 5-OP-RU loaded MR1-Tetramer. MAIT cells were sorted to a >98% purity using BD Influx (BD Biosciences). For some experiments, where specified, MAIT cells were further FACS sorted on the basis of CD69 expression to a purity of >97% for both CD69^-^ and CD69^+^ populations using BD Influx (BD Biosciences).

### Cell culture and viruses

ARPE-19 epithelial cells (ATCC) and ARPE-19-GFP cells that overexpress MR1 with GFP under the same promoter *via* a downstream internal ribosome entry sequence (38) were cultured in complete DMEM medium (DMEM with 4.5 g/L glucose and L-glutamine (Lonza), supplemented with 10% Foetal calf serum (FCS) (Sigma Aldrich) and 1% penicillin streptomycin (Gibco). A clinical VZV strain (VZV-S) and a recombinant VZV rOka-ORF10-GFP (VZV-GFP), which expresses GFP in fusion with ORF10 (39), were propagated in ARPE-19 cells in complete DMEM medium. All cells were cultured at 37 °C 5% CO_2_.

### VZV infection of immune cells and epithelial cells

PBMCs were either mock or VZV inoculated *via* co-culture with either uninfected or VZV infected ARPE-19 cells, respectively. The viral inoculum consisted of >75% VZV infected ARPE-19 cells demonstrating cytopathic effect (CPE). Inoculum was trypsinized, washed and resuspended in supplemented RPMI and added to PBMCs at a ratio of 1:2-5 ARPE-19: PBMC. For some experiments assessing viral infectivity, inoculum was added to PBMCs at various ratios of: 1:2, 1:5, 1:10, and 1:20 inoculum: PBMC. In parallel, inoculum was also added to ARPE-19 GFP expressing cells at identical ratios of 1:2, 1:5, 1:10 and 1:20 inoculum: ARPE-19-GFP cells for comparison. Kinetics of infection was investigated *via* a time-course experiment, inoculum was co-cultured with either PBMCs or ARPE-19-GFP cells at a ratio of 1:5 and harvested at various time-points of: 6, 24, 48 and 72 hours post inoculation. For experiments using total PBMCs, infections were performed in 12-well plates with 1-2 x 10^6^ PBMCs in 2 ml of complete RPMI medium per well. For experiments using FACS sorted MAIT cells, infections were performed in 24-well plates with 4 x 10^5^ cells in 600 ml complete RPMI medium per well. Following the addition of either mock or VZV infected cells to sorted MAIT cells and/or total PBMCs, cells were spinoculated in tissue culture plates for 15 minutes at 150 x *g* at 37°C. Plates were then incubated at 37°C 5% CO_2_ for 2 days.

### Antibodies

PBMCs for either surface staining flow cytometry or FACS sorting experiments were stained with the following fluorochrome conjugated antibodies: CD3-BUV395 (SK7), CD25-APC-H7 (M-A251), CD27-BUV661 (M-T271) (all BD Bioscience), CD8-SB780 (OKT8) (Thermo Fisher), CD4-PerCP/Cy5.5 (OKT4), CD56-BV605 (NCAM 16.2), TCR Vα7.2 (OF5A12), CCR4-BV421 (L291H4), CLA-AF647 (HECA-452), CCR2-APC (K036C2), CCR5-PE/Cy7 (J418F1), CCR6-BV421 (G034E3), CD69-BV421 (FN50), PD-1-PE/Dazzle (EH12.2H7), CD71-BV650 (CY1G4) (all Biolegend), VZV gE:gI (SG1-1, conjugated in house to Dy488), VZV gE:gI (SG1-1, conjugated in house to PE) (Meridian Life Sciences), 5-OP-RU loaded MR1 tetramer-PE, Ac-6-FP loaded MR1 Tetramer-PE. Matched isotype controls were used as negative controls.

### Flow cytometry

Cells were collected and viability stained with Live/Dead Blue (Invitrogen) as per manufacturer’s protocol. Cells were then resuspended and washed in FACS buffer, before staining with antibodies on ice for 45 minutes. Cells were washed in FACS buffer then fixed in 4.2% formaldehyde (BD Biosciences) at 4°C for 15 minutes before acquiring on a LSR-II cytometer (BD Biosciences).

### Flow cytometry data analysis

Data was analyzed using FlowJo software (versions 10.0.7 and 10.2; Tree Star). All PBMC data depicted was gated on live (as per the Live/Dead Blue viability dye staining) lymphocytes (as per distinct forward and side scatter morphology). MAIT cells were identified through positive co-staining with 5-OP-RU loaded MR1-Tetramer, anti-CD3 and anti-Vα7.2. All data observing viral infection of ARPE-19-GFP cells was gated on live (as per the Live/Dead Blue viability dye staining) GFP expressing cells.

### Infectious center assay

PBMCs were co-cultured with mock or VZV-ORF10-GFP infected ARPE-19 cells for 2 days. Cells were collected, stained and FACS sorted for CD3^+^ MR1-Tetramer^+^ Vα7.2^+^ (MAIT) cells. To remove extracellular virus, MAIT cells were washed in citrate buffer (40 mM C6H5O7Na3, 135 mM NaCl, 10 mM KCl [pH 3]), at room temperature for 2 minutes before washing in PBS (35, 40–42). In duplicate, MAIT cells (2.5x10^5^) were resuspended in complete RPMI medium and then added to pre-seeded ARPE-19 monolayers (7.5x10^5^) on glass coverslips in 24-well plates. Co-cultures were spinoculated at 15 minutes at 150 x *g* 37°C, before being incubated at 37°C 5% CO_2_ for 5 days to allow for formation of any CPE. Monolayers were fixed with 4.2% formaldehyde (BD Biosciences) at room temperature for 15 minutes, and then counterstained with DAPI. Imaging was performed using the Nikon Ti2-E Widefield fluorescence microscope.

### Statistical analyses

Statistical analyses were performed using GraphPad Prism (version 9; GraphPad Software).

### Ethics statement

All blood work was performed in accordance with The University of Sydney Human Research Ethics Committee approval. All blood donations were obtained under agreement with the Australian Red Cross Lifeblood service.

## Results

### Varicella Zoster Virus infects primary MAIT cells in human peripheral blood

**Figure 1:**
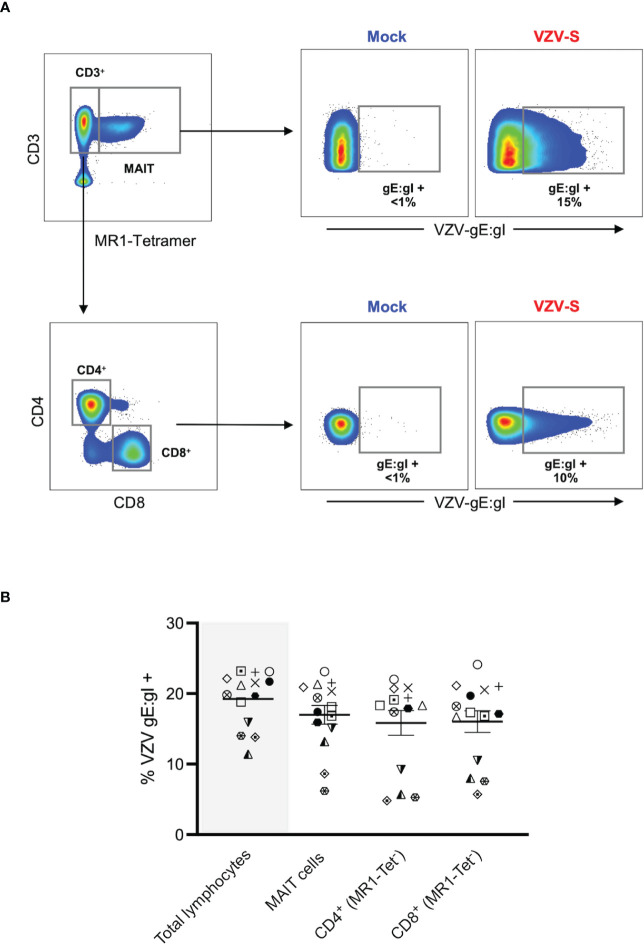
VZV infects MAIT cells from human peripheral blood. Human PBMCs were inoculated with mock or clinical VZV isolate (VZV-S) infected ARPE-19 epithelial cells for 2 days and then analyzed for infection by flow cytometry. **(A)** Representative flow cytometry plots depicting gating strategy of CD3^+^ MR1-Tetramer^+^ (MR1-Tet^+^) MAIT cells, non-MAIT (ie MR1-Tet^-^) CD3^+^ CD4^+^ (CD4 T cells) and non-MAIT (ie MR1-Tet^-^) CD3^+^ CD8^+^ cells (CD8 T cells), as well as quantifying surface VZV glycoprotein (g)E:gI expression on gated populations. **(B)** Frequencies of total live gE:gI^+^ lymphocytes (shaded), compared to MAIT cells, non-MAIT CD4^+^ and non-MAIT CD8^+^ cells (n=14). Symbols represent individual donors across the lymphocyte populations, with mean and standard error of mean (SEM) indicated by the bars. Statistical analysis between gE:gI expression on specific lymphocyte populations was performed *via* repeated measures (RM) one-way ANOVA with the Greenhouse-Geisser correction and Tukey’s multiple comparisons test.

Whilst the ability of VZV to infect human T cells is well documented (30, 31, 33, 34), the permissibility of MAIT cells to VZV is not known. To investigate this potential interaction, we assessed *via* flow cytometry the capacity of VZV clinical isolate (VZV-S) infected ARPE-19 epithelial cell-associated inoculum to infect MAIT cells in PBMCs. This cell-associated model of infection is commonly used in VZV studies (29, 30, 35) as VZV is very highly cell-associated *in vitro* (43). The mock and VZV infected inocula were excluded from analysis as per FSC-A SSC-A morphology gating and CD3 negative expression (**Figure 1**). The detection of the surface VZV glycoprotein (g)E:gI complex, which is expressed late in the VZV replicative cycle, was utilized as a means to detect virally infected cells (33, 35–37, 44, 45). Therefore, all results presented within this study that refer to the “VZV^+”^ or “VZV infected” are expressing VZV gE:gI.

MAIT cells were identified *via* co-staining of 5-OP-RU loaded MR1 tetramer and CD3 (**Figure 1A**), with a vast majority of that population positively staining for the canonical MAIT TCR, Vα7.2 (range: 96.2-99.8%) (**Supplementary Figure 1A**). In line with previous studies (1, 2), we detected an average of 2.4% of live PBMCs as MAIT cells, with no difference in the frequency of live MAIT cells between mock and VZV inoculated samples (**Supplementary Figure 1B**). When examining the transfer of viral infection to the total pool of live PBMCs, a mean of 19.2% (range 11.4-23.2%) of these cells were VZV gE:gI^+^ (**Figure 1B**).

A mean of 17% of MAIT cells were gE:gI^+^ (range 6.2-23.2%). When examining gE:gI expression in non-MAIT cell (ie MR1 Tet^-^) CD4^+^ cells (mean 15.8%, range 4.8-22%) and non-MAIT cell (ie MR1-Tet^-^) CD8^+^ cells (mean 16%, range 5.7-24.1%) populations, we observed no significant difference in infection level when compared to MAIT cells across 14 different donors (**Figure 1B**). Furthermore, gE:gI expression on MAIT cells was detected as early as 6 hours post inoculation (mean 4.7%, range 3.6-5.2%) and peaked at 48 hours post inoculation (mean 23%, range 20.5-25%) (**Supplementary Figure 2A**). Additionally, gE:gI expression was detected on MAIT cells at various viral inoculum: PBMC ratios from 1:2 (mean 21.7%, range 20.5-22.6%) to 1:20 (mean 7.7%, range 5-12.1%) (**Supplementary Figure 2B**). In a comparison to epithelial (ARPE-19) cells, MAIT cells were less permissive to VZV infection (**Supplementary Figure 2**). Together, these results identify MAIT cells as a T lymphocyte compartment that is permissive to VZV infection. Furthermore, VZV infection of MAIT cells was less than that of infection of ARPE-19 cells but comparable to infection of non-MAIT cell CD4^+^ and CD8^+^ T lymphocyte populations.

### VZV infects diverse MAIT cell subsets

**Figure 2:**
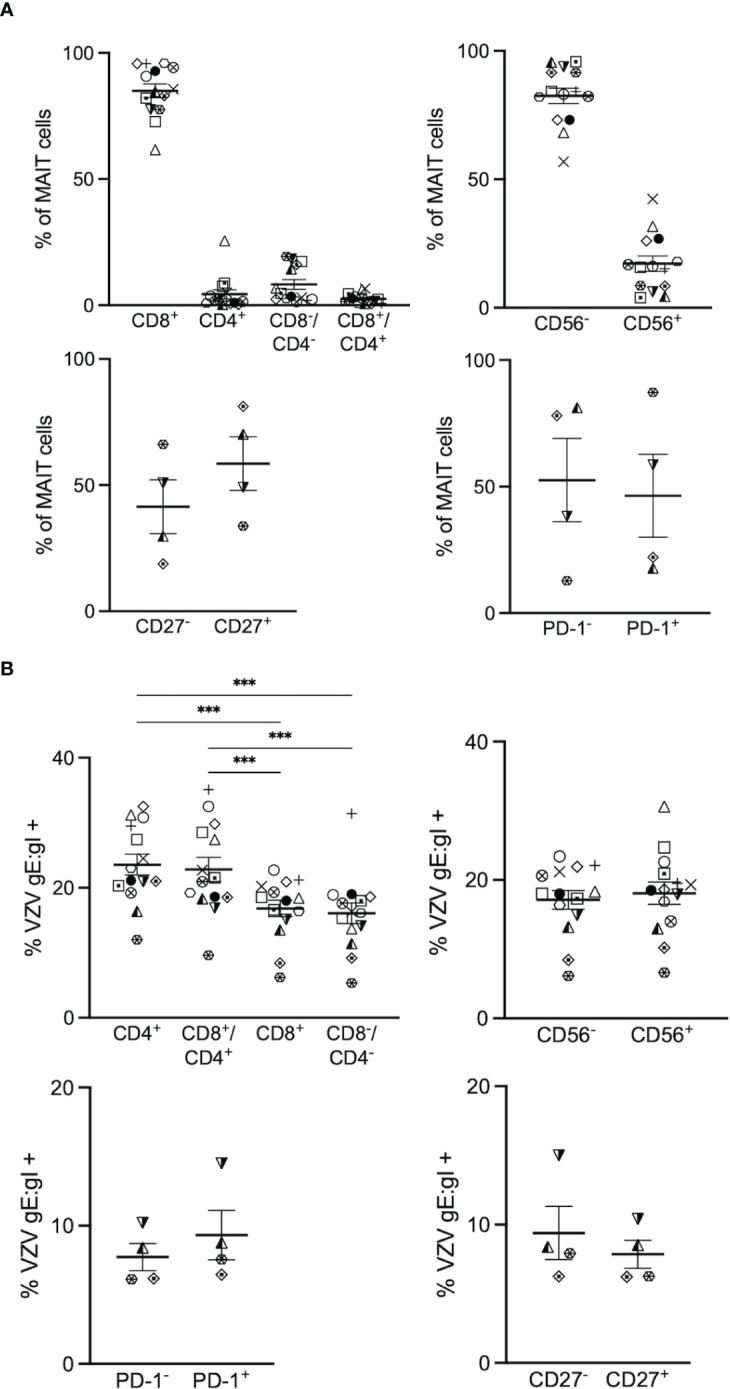
VZV infects diverse MAIT cell subsets. Human PBMCs were inoculated with mock or VZV-S infected ARPE-19 epithelial cells for 2 days and then analyzed for infection by flow cytometry as per surface VZV-gE:gI expression **(A)** Graphs showing frequencies of various MAIT cell subpopulations (n=4-14). **(B)** Frequencies of gE:gI^+^ lymphocytes within each subpopulation depicted, with symbols representing individual donors across the MAIT subpopulations, with mean and SEM indicated by the bars. Statistical analysis of VZV gE:gI expression, comparing CD4^+^ cells with CD8^+^ cells, and CD4^+^/CD8^+^ cells compared to CD4^-^/CD8^-^ cells was performed *via* RM one-way ANOVA with the Greenhouse-Geisser correction and Tukey’s multiple comparisons test (n=14).***p<0.001. Statistical analysis of gE:gI expression on MAIT cells expressing CD56^-^ was compared to those expressing CD56^+^ (n=14), CD27^-^ compared to CD27^+^ (n=4) and PD-1^-^ compared to PD-1^+^ (n=4) was performed *via* two tailed paired *t* test.

Similar to other innate-like T cell populations, the MAIT cell compartment consists of a heterogenous mix of subpopulations with distinct functional attributes (1, 46, 47). Using flow cytometry, we sought to determine the extent to which VZV infection of MAIT cells was associated with different subsets of MAIT cells. MAIT cells were split into distinct subpopulations based on the expression of the following cell surface markers: co-receptor (CD8 and CD4), co-stimulatory (CD27), immune checkpoint marker (PD-1), and CD56. Flow cytometric analysis revealed the frequencies of MAIT cell sub-populations (**Figure 2A**), and these were consistent with previous literature (1, 46–48). Furthermore, no change of frequencies within MAIT cell subpopulations between mock and VZV infected samples was observed (**Supplementary Figure 1C**).

When examining the co-receptor subpopulations, there was a significantly higher level of infection of CD4^+^ (mean 23.6%, range 12-32.5%) and double positive (CD4^+^/CD8^+^) (mean 22.8%, range 9.6-35.1%) MAIT cells compared to CD8^+^ (average 16.8%, range 6.2-22.7%) and double negative (CD4^-^/CD8^-^) (mean 16%, range 5.4- 31.4%) MAIT cells (**Figure 2B**). There was no significant difference in the level of infection between CD56^+^ (mean 18.1%, range 6.6-30.6%) and CD56^-^ (mean 17.1%, range 6.1-23.4%) MAIT cells (**Figure 2B**). Furthermore, there was no significant difference in the level of infection between CD27^+^ (mean 7.9%, range 6.2-10.4%) and CD27^-^ (mean 9.4%, range 6.3-15%) MAIT cells, and both PD-1^+^ (mean 9.3%, range 6.5-14.5%) and PD-1^-^ (mean 7.7%, range 6.1-10.2%) MAIT cells demonstrated similar levels of infection (**Figure 2B**). Overall, all subpopulations examined in this study were comparably infected, with the exception of the proportion of CD4^+^ and CD8^+^/CD4^+^ double positive MAIT cells being associated most with VZV infection.

### VZV infection of MAIT cells is associated with increased CD69 and CD71 expression

**Figure 3:**
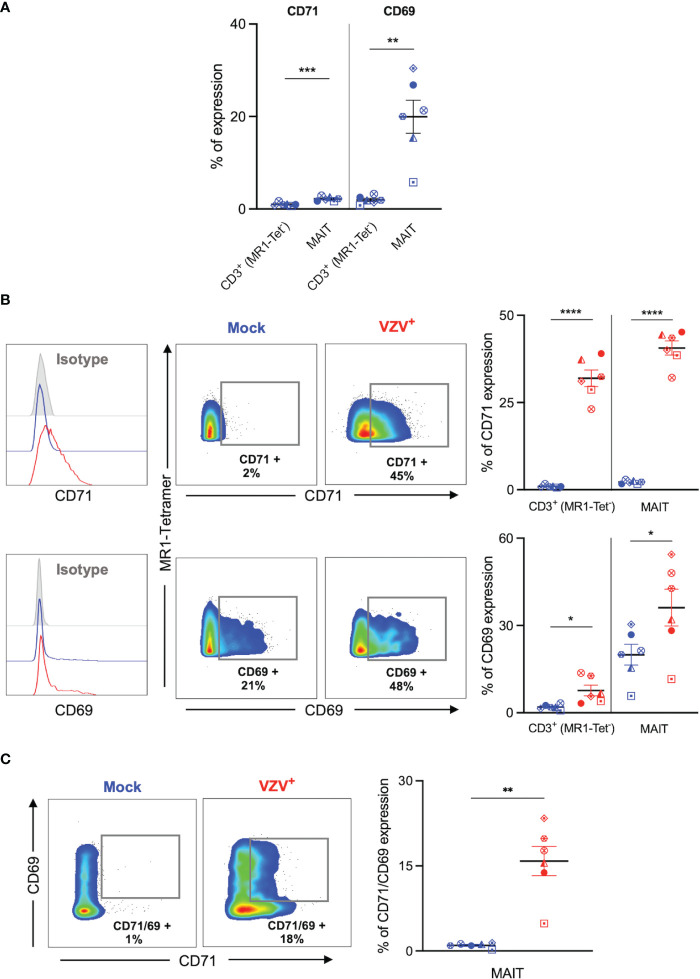
VZV infection of MAIT cells is associated with expression of early activation and proliferation markers. Human PBMCs were inoculated with mock or VZV-S infected ARPE-19 epithelial cells for 2 days and then analyzed for infection (gE:gI), proliferation (CD71), and early activation (CD69) markers by flow cytometry. **(A)** Graph shows comparative frequency of surface CD71 and CD69 expression in mock inoculated non-MAIT (ie MR1-Tet^-^) CD3^+^ T cells and MAIT cell populations, with symbols representing individual donors (n=6). Statistical analysis was performed *via* two tailed paired *t* test. **p<0.001, ***p<0.001. **(B)** Representative histograms show expression of CD71 and CD69 by MAIT cells for Mock (blue) and VZV infected (VZV^+^) (red) populations corresponding to their respective isotype controls (filled grey). Flow cytometry plots show surface expression of CD71 and CD69 on MAIT cells for Mock (blue) and VZV infected (VZV^+^) (red) populations. Graphs show frequency of CD71 and CD69 in non-MAIT (ie MR1-Tet^-^) CD3^+^ and MAIT cell subpopulations, with symbols representing individual donors, and mean and SEM indicated by the bars. Statistical analysis of CD71 and CD69 expression between Mock and VZV^+^ infected non-MAIT CD3^+^ cells and MAIT cells was performed *via* two tailed paired *t* test (n=6). *p<0.05, ****p<0.0001. **(C)** Flow cytometry plots show CD69 *vs* CD71 double expression on Mock (blue) and VZV infected (VZV^+^) (red) populations. Graph shows frequency of CD69/CD71 double expressing MAIT cells, with symbols representing individual donors, and mean and SEM indicated by the bars. Statistical analysis of CD71 and CD69 expression between Mock and VZV^+^ infected non-MAIT CD3^+^ cells and MAIT cells was performed *via* two tailed paired *t* test (n=6). *p<0.05, ****p<0.0001.

We sought to determine whether VZV infection of MAIT cells was associated with an altered expression profile of activation (CD69) and proliferation (CD71) markers, as determined by flow cytometry of mock and VZV infected MAIT cells. As an additional comparison we also examined mock and VZV infection of non-MAIT (ie MR1-Tet^-^) CD3^+^ T cells. Mock inoculated MAIT cells endogenously expressed higher CD71 (mean 1%, SEM +/- 0.15%) compared to non-MAIT CD3^+^ T cells (mean 2.18%, SEM +/- 0.19%). Whilst a significantly greater proportion of mock MAIT cells endogenously expressed higher CD69 (mean 1.95%, SEM +/- 0.35%) compared to non-MAIT CD3^+^ T cells (mean 19.95%, SEM +/- 3.56%) (**Figure 3A**). Furthermore, our analysis revealed a significantly higher proportion of VZV infected (gE:gI^+^) MAIT cells expressed CD71 (mean 40.62, SEM +/- 2.01) compared to mock infected (mean 2.18%, SEM +/- 0.19%) (**Figure 3B**). This was also the case when observing CD69 expression in VZV infected MAIT cells (mean 36.15%, SEM +/- 6.32%) compared to mock (mean 19.95%, SEM +/- 3.56%) (**Figure 3B**). Indeed, a significantly greater number of CD71/CD69 double positive MAIT cells was correspondingly observed in the VZV infected condition (mean 15.84%, SEM +/- 2.59%) compared to mock (mean 0.99%, SEM +/- 0.17%) (**Figure 3C**). Furthermore, a higher proportion of CD69 and CD71 expressing cells were similarly detected in VZV infected non-MAIT CD3^+^ T cells compared to mock infected counterparts (**Figure 3B**). When analysing the VZV bystander (gE:gI^-^) MAIT cell subpopulation, only a conservative albeit significant increase of CD71 expression compared to mock MAIT cells was observed (mean 4.58%, SEM +/- 0.1%), whilst no significant change of CD69 expression compared to mock was detected (**Supplementary Figure 4A**).

Following on previous reports that demonstrate a preferential infection of CD69 expressing T lymphocytes (30) we FACS sorted MAIT cells by CD69 expression into two populations: CD69^-^ and CD69^+^ MAIT cells (**Supplementary Figure 3A**). We observed no significant difference in VZV infection when comparing CD69^-^ and CD69^+^ sorted MAIT cells (**Supplementary Figure 3B**). We also examined CD69 expression on these MAIT cells sorted on the basis of CD69 expression. In comparison to mock infected counterparts, we did not observe an increase of CD69 expression by CD69-sorted MAIT cells following VZV infection, however, there was a significant increase in CD69 expression following VZV infection of CD69^+^ sorted MAIT cells (**Supplementary Figure 3C**). Collectively, these data indicate that VZV infection of MAIT cells and non-MAIT T cells is associated with the upregulation of CD71^+^ and CD69^+^ expression.

### VZV infected MAIT cells maintain a highly expressed extravasation and skin homing program

**Figure 4:**
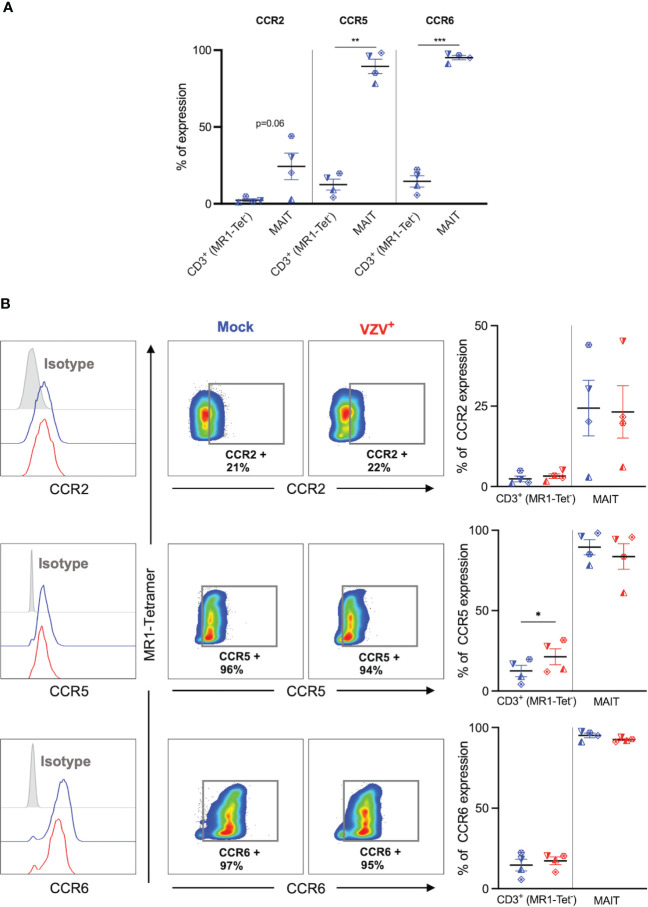
VZV infection of MAIT cells does not suppress CCR2, CCR5 and CCR6 expression. Human PBMCs were inoculated with mock or VZV-S infected ARPE-19 epithelial cells for 2 days and then analyzed for infection (gE:gI), and extravasation chemokine receptor markers (CCR2, CCR5 and CCR6) by flow cytometry. **(A)** Graph shows comparative frequency of surface CCR2, CCR5, and CCR6 expression in mock inoculated non-MAIT (ie MR1-Tet^-^) CD3^+^ T cells and MAIT cell populations, with symbols representing individual donors (n=4). Statistical analysis was performed *via* two tailed paired *t* test. **p<0.001, ***p<0.001. **(B)** Representative histograms show expression of CCR2, CCR5 and CCR6 by MAIT cells for Mock (blue) and VZV infected (VZV^+^) (red) populations corresponding to their respective isotype controls (filled grey). Flow cytometry plots show surface expression of CCR2, CCR5 and CCR6 on mock infected (blue) and VZV infected (red) MAIT cell populations. Graphs show frequencies of non-MAIT CD3^+^ T cells and MAIT cells expressing CCR2, CCR5 and CCR6, with symbols representing individual donors, with mean and SEM indicated by the bars. Statistical analysis of CCR2, CCR5 and CCR6 expression between Mock and VZV^+^ infected non-MAIT CD3^+^ cells and MAIT cells was performed *via* two tailed paired *t* test (n=4). *p<0.05.

We next sought to determine the impact of VZV infection on the natively high expression of extravasation and skin homing markers on MAIT cells (9, 10). Initially, expression of key extravasation markers CCR2, CCR5 and CCR6 on mock inoculated non-MAIT (ie MR1-Tet^-^) CD3^+^ cells was compared to mock infected MAIT cells. This analysis revealed that a greater proportion of MAIT cells endogenously expressed CCR5, CCR6 and CCR2 (**Figure 4A**), which is consistent with MAIT cells possessing a potent program for extravasastion (9). In the context of VZV infection of MAIT cells, we observed no significant difference in proportion of infected cells expressing CCR2, CCR5 or CCR6 in comparison to mock infection (**Figure 4B**). The proportion of non-MAIT CD3^+^ T cells expressing CCR2 and CCR6 were also not different between mock and VZV infection, whereas there was a significant increase in the proportion of non-MAIT CD3^+^ T cells expressing CCR5 in VZV infection (mean 21.3%, SEM +/- 4.94%) when compared to mock infected counterparts (mean 12.5, SEM +/- 3.56%) (**Figure 4B**).

**Figure 5:**
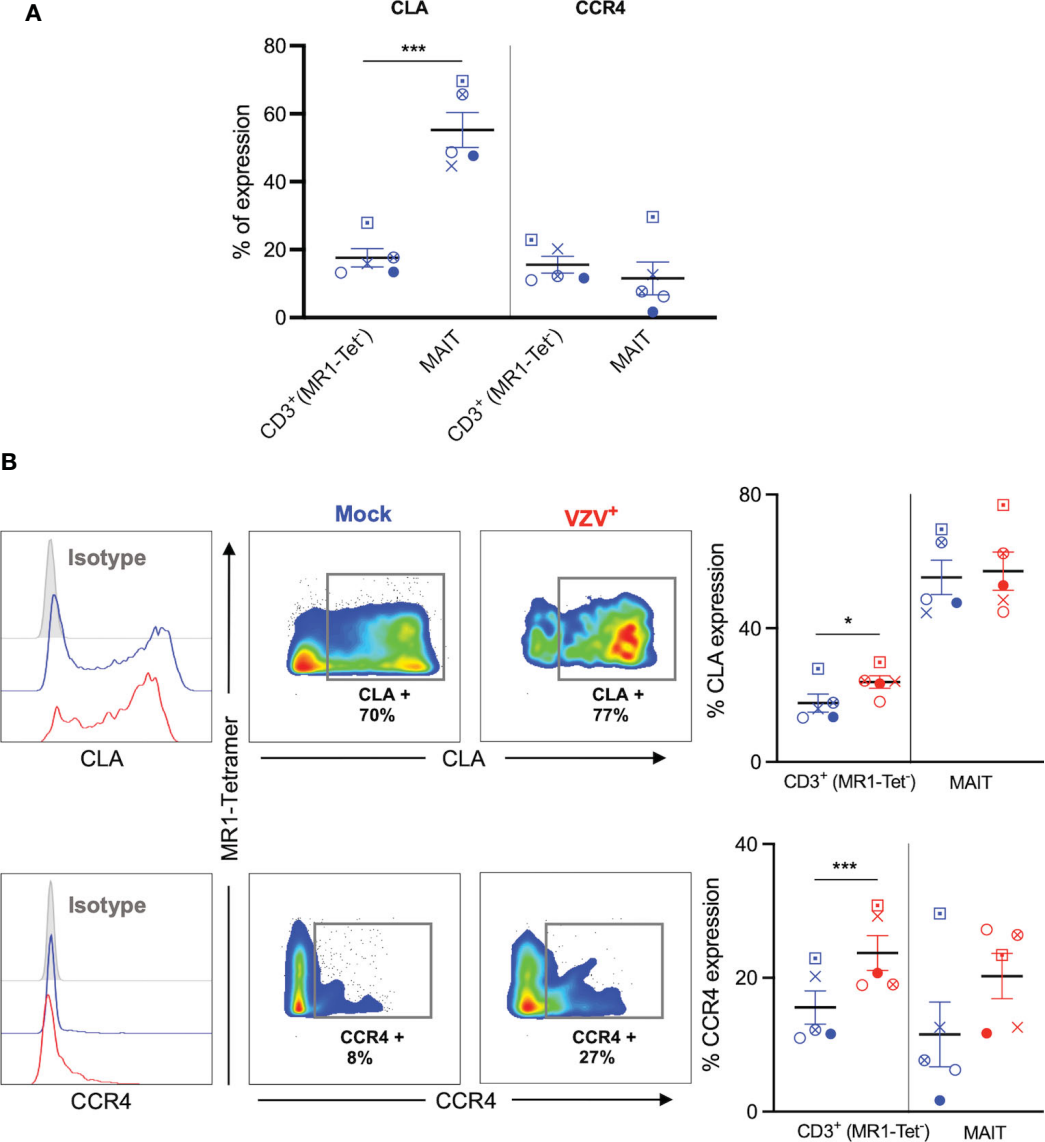
VZV infection of MAIT cells retains expression of CLA and CCR4 skin homing chemokine receptor expression. Human PBMCs were inoculated with mock or VZV-S infected ARPE-19 epithelial cells for 2 days and then analyzed for infection (gE:gI), and skin homing markers (CLA and CCR4) by flow cytometry. **(A)** Graph shows comparative frequency of surface CLA, and CCR4 expression in mock inoculated non-MAIT (ie MR1-Tet-) CD3^+^ T cells and MAIT cell populations, with symbols representing individual donors (n=5). Statistical analysis was performed *via* two tailed paired *t* test. ***p<0.001. **(B)** Representative histograms show expression of CLA and CCR4 by MAIT cells for Mock (blue) and VZV infected (VZV^+^) (red) populations corresponding to their respective isotype controls (filled grey). Flow cytometry plots from one donor show surface expression of CLA and CCR4 on MAIT cells for Mock (blue) and VZV infected (VZV^+^) (red) populations. Graphs show frequencies of non-MAIT CD3^+^ and MAIT cells expressing CLA and CCR4 with symbols representing individual donors across the subpopulations, with mean and SEM indicated by the bars. Statistical analysis of CLA and CCR4 expression between Mock and VZV^+^ infected non-MAIT CD3^+^ and MAIT cells was performed *via* two tailed paired *t* test (n=5). *p<0.05, ***p<0.001.

The upregulation of skin homing markers such as CLA and CCR4 has been reported on VZV infected tonsillar T cells (30, 33, 34), providing impetus to examine the expression of these skin homing markers on VZV infected MAIT cells. A high proportion of mock infected MAIT cells expressed CLA compared to non-MAIT CD3^+^ cells (**Figure 5A**), which is consistent with previous literature (10). In contrast, the proportion of both uninfected MAIT cells and non-MAIT CD3^+^ cells that expressed CCR4 was relatively low, and comparable to each other (**Figure 5A**).

There was no significant difference between mock and VZV infected in the proportion of CLA^+^ MAIT cells, whilst VZV infected non-MAIT CD3^+^ cells demonstrated a significant upregulation of CLA compared to mock infected non-MAIT CD3^+^ cells (**Figure 5B**). Furthermore, there was a trend to an increased proportion of CCR4^+^ expression in VZV infected MAIT cells compared to mock (**Figure 5B**), whilst no change was observed in VZV bystander MAIT cells compared to mock (**Supplementary Figure 4B**). In addition, a greater proportion of CCR4^+^ non-MAIT CD3^+^ cells was observed during VZV infection compared to mock (**Figure 5B**). These data demonstrate that VZV infection of MAIT cells does not impair CLA or CCR4 expression and that VZV infection of non-MAIT CD3^+^ cells increases the expression of these skin homing markers.

Together, these results indicate that VZV infection of MAIT cells does not impair, but rather maintains expression of cell-surface cellular proteins associated with extravasation and skin homing programs.

### MAIT cells support *de-novo* viral replication and virus transmission

**Figure 6:**
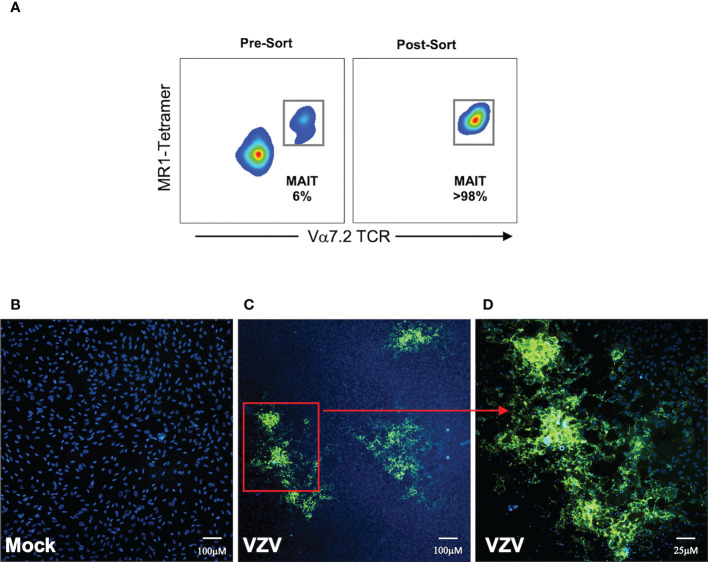
MAIT cells support *de-novo* viral replication and virus transmission. Human PBMCs were inoculated with mock or a GFP-tagged VZV infected ARPE-19 epithelial cells for 2 days and then FACS sorted for MAIT cells (CD3^+^ MR1-Tetramer^+^ Vα7.2^+^). **(A)** Representative flow cytometry plot depicts MAIT cell frequencies of total live lymphocytes pre- and post-sorting. **(B, C)** Sorted mock or VZV-GFP exposed MAIT cells were citrate buffer washed three times and then added to ARPE-19 epithelial cell monolayers at a ratio of 1 sorted MAIT cell to 5 epithelial cells. Co-cultures were incubated at 37°C 5% CO_2_ and five days later monolayers were fixed, counterstained with DAPI and infectious centers visualized by the detection of VZV-GFP by fluorescent microscopy. Representative images (from four replicate experiments) of ARPE-19 monolayers exposed to **(B)** mock infected MAIT cells or **(C)**, inset in **(D)** VZV-GFP exposed MAIT cells are shown.

Having established that MAIT cells were infected with VZV, we sought to determine whether VZV infected MAIT cells were capable transmitting infectious virus to other cells. We performed an infectious center assay which has been previously utilized to demonstrate productive infection and new infectious virion production in VZV infected T cells, NK cells and dendritic cells (29, 30, 35). MAIT cells were isolated by FACS sorting from PBMCs that had been exposed to a GFP-tagged VZV (VZV-ORF10-GFP) or had been mock infected (**Figure 6A**). The sorted MAIT cells were washed with citrate buffer to inactivate and detach any surface bound virions (41, 42, 49), before being co-cultured with uninfected ARPE-19 epithelial cell monolayers. After five days in culture, the presence of virus-induced cytopathic effect (CPE) in the ARPE-19 monolayer was determined *via* detection of GFP signal by fluorescence microscopy (**Figures 6C, D**). ARPE-19 cell monolayers co-cultured with Mock infected MAIT cells yielded no GFP fluorescence (**Figure 6B**) whereas distinct GFP^+^ infectious centers were readily detected in ARPE-19 monolayers co-cultured with MAIT cells infected with VZV (**Figures 6C, D**). These data demonstrate that that human MAIT cells infected with VZV are capable of transmitting infectious virus to epithelial cells.

## Discussion

A hallmark of VZV primary infection is the dissemination of cell-associated virus in infected individuals (27). Uncovering the full repertoire of immune cell populations that VZV infects is crucial in forming a better understanding of the key pathogen-host interactions that result in the widespread manifestation of cutaneous vesicles and subsequent inter-host transmission of virus (27, 50, 51). In this study, we provide evidence that VZV productively infects blood-derived MAIT cells, with a consequence being a capacity to transmit infectious virus to epithelial cells. We also demonstrate that VZV infected MAIT cells display a modulated activation status whilst retaining a highly expressed extravasation and skin homing program.

Cell-associated VZV infection of human PBMCs revealed infection of MAIT cells that was at a similar magnitude to non-MAIT CD4^+^ and CD8^+^ T cells. Furthermore, VZV infection did not alter the overall viability of MAIT cells. This is consistent with *in vitro* studies which reported no significant loss of viability of VZV infected T cells, NK cells or DCs (29–31, 35, 52), but contrasts with the rapid death of MAIT cells during *in vitro* infection by measles virus (26).

A recent study uncovered Siglec-7 (CD328) as a key cell receptor that binds to VZV entry glycoprotein (g)B and mediates the entry of VZV in monocytes (53, 54). However, Siglecs are poorly expressed in human T cells due to their potentially negative regulation of TCR signalling (55–57). Whilst the lack of Siglec-7 expression by T cells may potentially explain the higher level of infection observed in monocytes (45, 52–54), it also suggests a potentially distinct T lymphocyte specific entry receptor through which VZV gains entry.

Despite the extraordinary level of conservation present within the MR1-MAIT cell axis, there is a growing understanding of phenotypic and functional heterogeneity present within circulating MAIT cells. Indeed, MAIT cell expression of NK cell associated markers such as CD56 is associated with a greater propensity for response to cytokine stimulation (46). We found no preference of VZV infection across MAIT cells either expressing or non-expressing CD56, CD27 or PD-1, nor did we observe frequency alterations of the subpopulations studied. Whilst we did demonstrate a significantly higher proportion of infection within CD4^+^ and CD4^+^/CD8^+^ MAIT cells, the biological impact of this is not clear given they represent a small subset of the overall MAIT cell compartment (1).

Interestingly, analysis of the activation marker CD69, revealed that VZV infection was associated with a significantly higher proportion of CD69^+^ MAIT cells compared to mock infection in both the context of whole PBMCs and MAIT cells FACS sorted on the basis of CD69 expression. These finding are similar to earlier studies that demonstrate an upregulation of CD69 in tonsillar T cells as a consequence of VZV infection (33, 34, 58, 59). This is similar with several lymphotropic viruses as they also require T cell activation for productive infection. Indeed, HIV-1 infection of resting naïve CD4^+^ T cells results in an abortive non-replicative infection, whilst either mitogenic or anti-CD3/CD28 mediated stimulation of T cells drives production of replicating virus (60–62). Similarly, viral replication is increased when either VZV infected T cells or NK cells are treated with stimuli such as PMA or IL-2 respectively (30, 35). Like CD69, the proportion of MAIT cells expressing the T cell proliferation marker CD71 was significantly higher during VZV infection compared to mock infection, although we did not observe an overall difference in the number of MAIT cells between mock and VZV infected cultures. As CD71 plays a role in initiating proliferation and activation in resting, quiescent or terminally differentiated lymphocytes through permitting and accommodating for an increased metabolic demand (63–69), the upregulation of CD71 may rather reflect the metabolically higher demand of generating viral progeny.

Upregulation of skin homing markers on VZV infected T cells and NK cells has been reported (30, 33–35), yet there are no studies that observe markers of extravasation potential by VZV infected lymphocytes; a crucial step lymphocytes must take before accessing skin sites. In light of work by Lee et al., 2018 which elegantly demonstrated a highly expressed extravasation program by MAIT cells (9), we determined if VZV infected MAIT cells are likely to retain this potential. Similar to previous reports, MAIT cells endogenously expressed greater levels of CCR2, CCR5 and CCR6 compared to non-MAIT CD3^+^ cells (9). Importantly, VZV infected MAIT cells were able to retain the constitutively high expression of CCR2, CCR5 and CCR6. Under homeostatic conditions, the skin homing capacity of circulating T cells is tightly regulated, whilst chronic skin conditions such as psoriasis are characterized by infiltrating T cells with markedly increased levels of CCR4 and CLA expression (70, 71). We found that VZV infected MAIT cells maintained a pronounced expression of CLA along with a trend to upregulated CCR4 expression. Studying the skin homing capacity of VZV infected MAIT cells *in vivo* is challenging given the high-species specificity of this virus and the lack of an animal model to study productive infection and MAIT cells (27). Migration assays utilizing a cognate skin homing chemokine such as CC chemokine ligand (CCL) 17 would illuminate whether VZV infected MAIT cells have a functional migration capacity, as shown by VZV infected tonsillar T cells (31).

The clinical manifestation of varicella, with widespread cutaneous lesions suggests a requirement for transfer of virus from the infected lymphocyte carrier to target keratinocytes (27, 72). The formation of distinct infectious centers in epithelial cell monolayers after incubation with VZV infected MAIT cells highlights a previously unknown target immune cell sub-population that may enable such infectious virus transfer from circulating cells to cutaneous sites. Whilst VZV is able to maintain and upregulate skin homing markers on NK cells, they remain functionally incapacitated to stimulation (35, 36). Whether VZV infection of MAIT cells represents a strategy for virus dissemination whilst also modulating MAIT cell effector functions such as cytokine production and cytolytic activity, and/or whether MAIT cells exert any VZV effector function remain important areas of investigation, particularly in the context of emerging evidence of a role for MAIT cells in a range of other virus infections (21–25).

Our understanding of the range of immune cell populations that VZV infects and manipulates to achieve inter-host dissemination is becoming increasingly diverse. Here we describe an immune cell subset permissive to VZV infection and propose MAIT cells to be a crucial target during VZV infection that contributes to the dissemination of virus.

## Data availability statement

The raw data supporting the conclusions of this article will be made available by the authors, without undue reservation.

## Ethics statement

The studies involving human participants were reviewed and approved by University of Sydney Human Research Ethics Committee. Written informed consent for participation was not required for this study in accordance with the national legislation and the institutional requirements.

## Author contributions

SP performed experiments. BS, AA and SP conceived the study and planned experiments. AC provided key reagents. All authors contributed to the article and approved the submitted version.
