# Supplementary figures and images for: Varicella Zoster Virus infects mucosal associated Invariant T cells
Source: Front Immunol. 2023 Mar 17;14:1121714. doi: 10.3389/fimmu.2023.1121714 (PMC10063790; doi:10.3389/fimmu.2023.1121714)

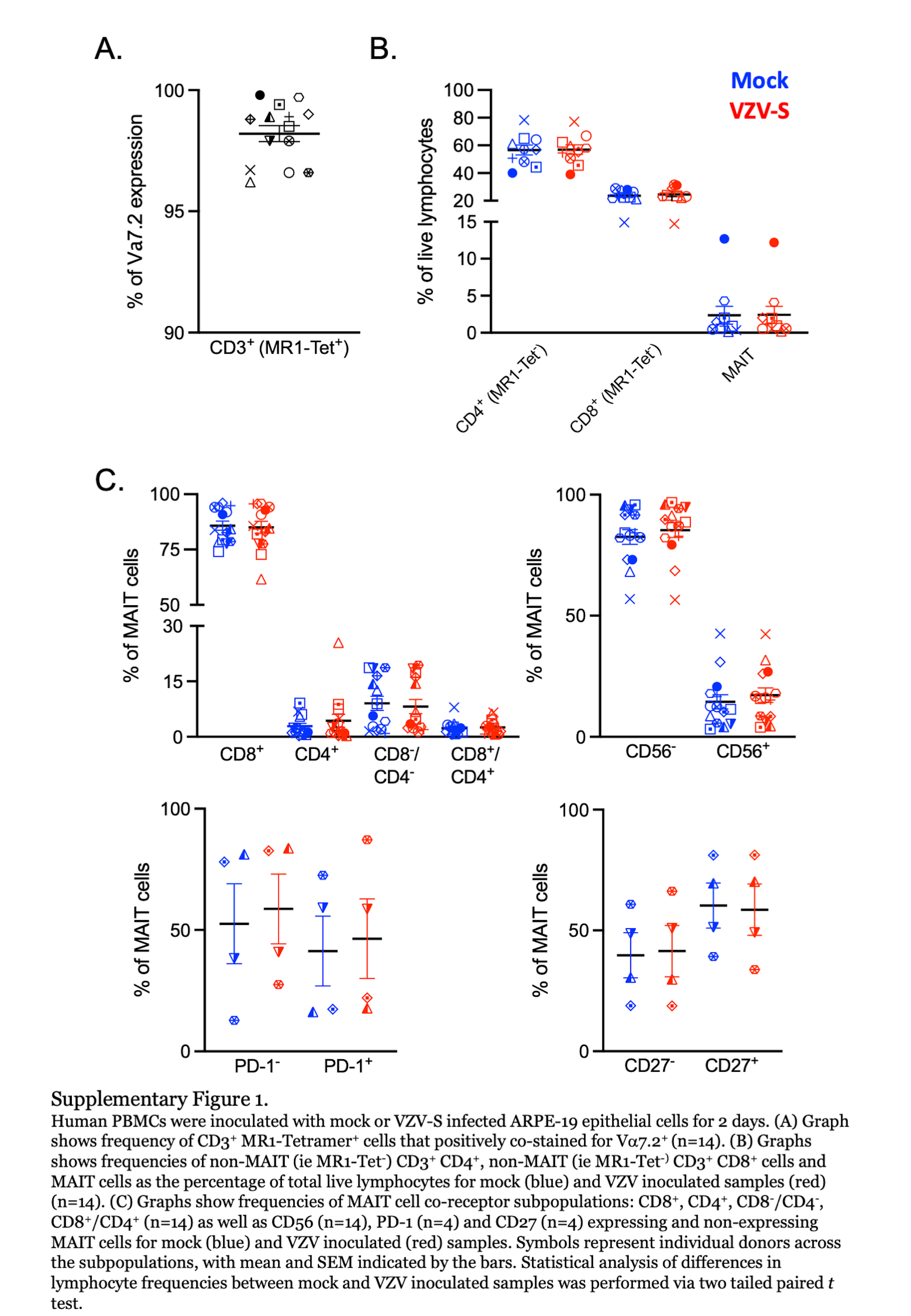

Supplement: Supplementary file 1 [file Image_1.jpg]

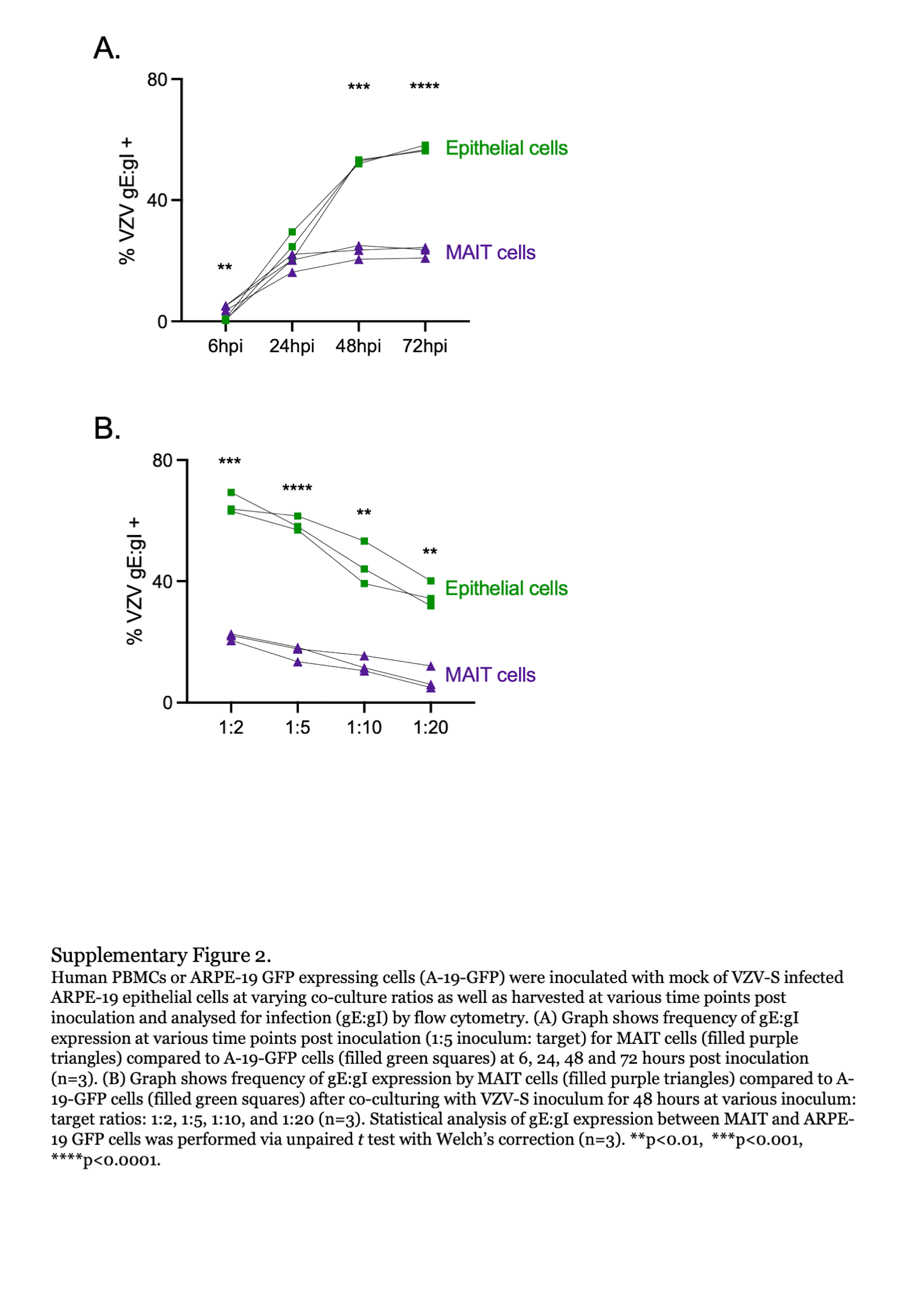

Supplement: Supplementary file 2 [file Image_2.jpg]

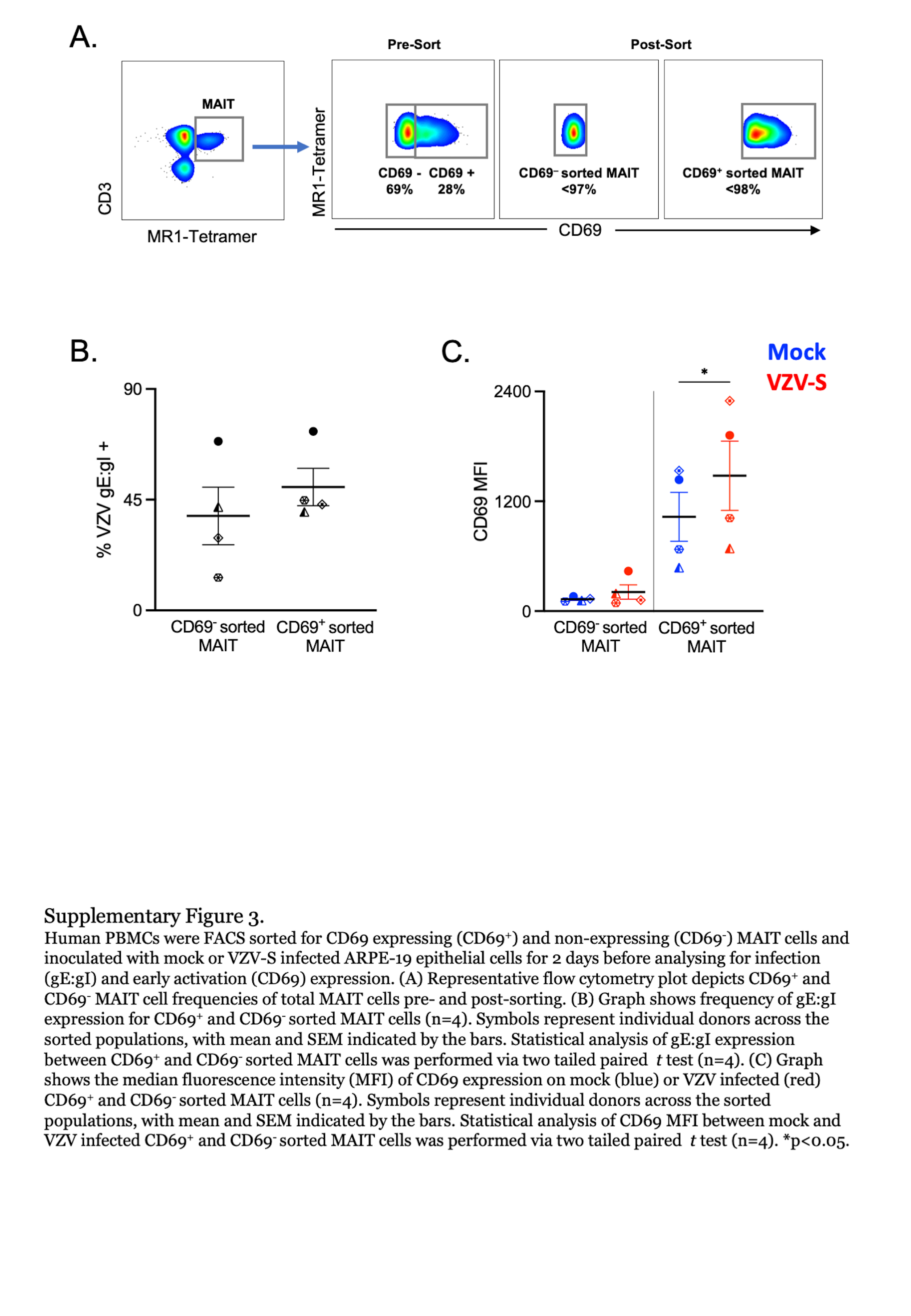

Supplement: Supplementary file 3 [file Image_3.jpg]

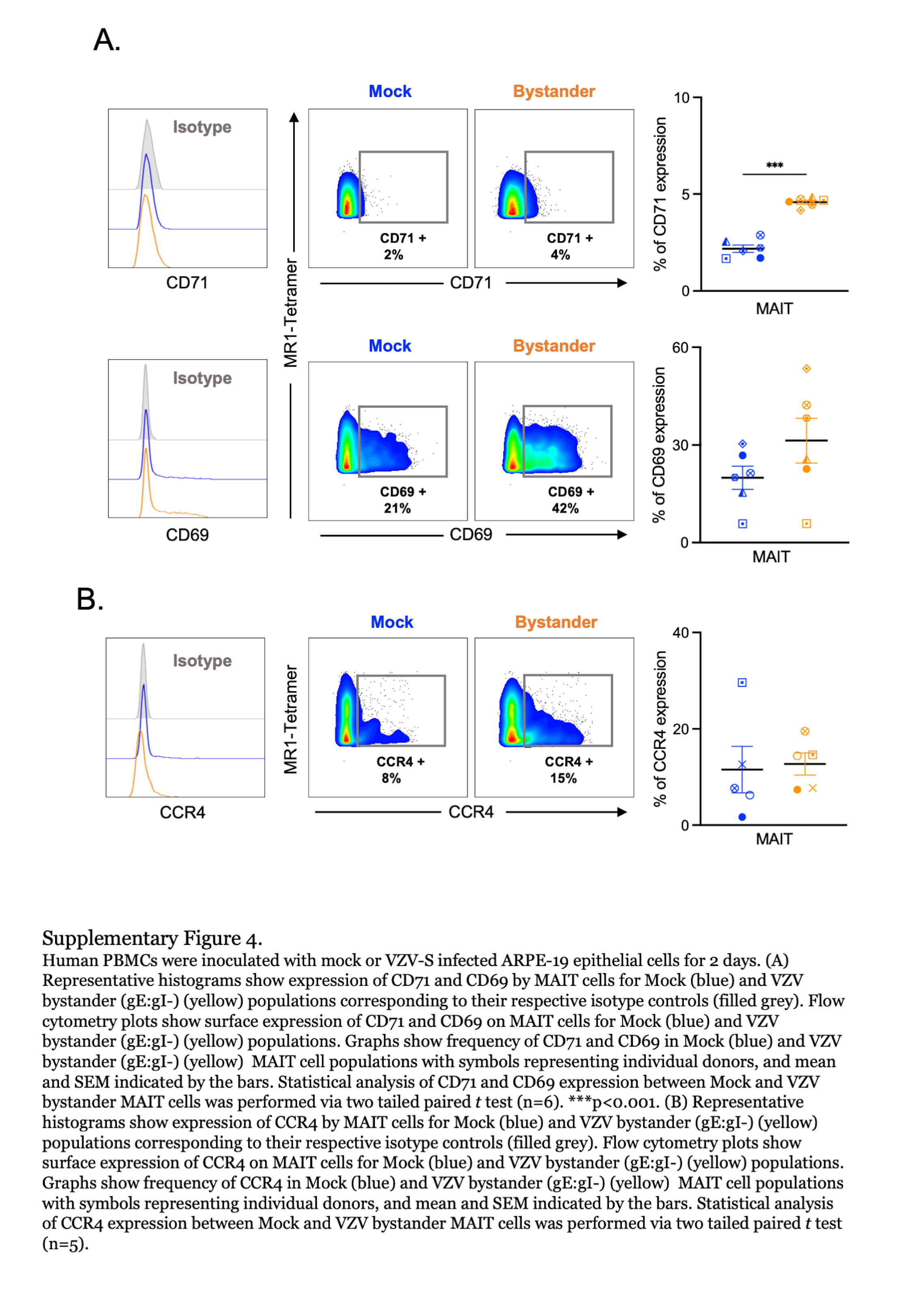

Supplement: Supplementary file 4 [file Image_4.jpg]
